# Effects of Strontium-Doped β-Tricalcium Scaffold on Longitudinal Nuclear Factor-Kappa Beta and Vascular Endothelial Growth Factor Receptor-2 Promoter Activities during Healing in a Murine Critical-Size Bone Defect Model

**DOI:** 10.3390/ijms21093208

**Published:** 2020-05-01

**Authors:** Mersedeh Tohidnezhad, Yusuke Kubo, Philipp Lichte, Tobias Heigl, Diana Roch, Nazanin Barahmand Pour, Christian Bergmann, Tolga Taha Sönmez, Jennifer Vanessa Phi Hock, Athanassios Fragoulis, Felix Gremse, Stefanie Rosenhain, Alexander Slowik, Michaela Bienert, Nisreen Kweider, Christoph Jan Wruck, Holger Jahr, Frank Hildebrand, Hans Christoph Pape, Sabine Neuß, Horst Fischer, Thomas Pufe

**Affiliations:** 1 Department of Anatomy and Cell Biology, RWTH Aachen University Hospital, 52074 Aachen, Germany; ykubo@ukaachen.de (Y.K.); tobias.heigl@kuleuven.be (T.H.); dianaroch23@gmail.com (D.R.); nbarahmandp@gmail.com (N.B.P.); Taha.Soenmez@klinikum-karlsruhe.de (T.T.S.); jennyhock91@gmail.com (J.V.P.H.); afragoulis@ukaachen.de (A.F.); nkweider@ukaachen.de (N.K.); cwruck@ukaachen.de (C.J.W.); hjahr@ukaachen.de (H.J.); tpufe@ukaachen.de (T.P.); 2 Department of Trauma Surgery, RWTH Aachen University Hospital, 52074 Aachen, Germany; plichte@ukaachen.de (P.L.); fhildebrand@ukaachen.de (F.H.); hans-christoph.pape@usz.ch (H.C.P.); 3 Department of Dental Materials and Biomaterials Research, RWTH Aachen University Hospital, 52074 Aachen, Germany; Christian_JD_Bergmann@gmx.de (C.B.); hfischer@ukaachen.de (H.F.); 4 Department of Oral, Cranio-Maxillofacial and Facial Plastic Surgery, Hospital Karlsruhe of University Freiburg, 76133 Karlsruhe, Germany; 5 Institute for Experimental Molecular Imaging, RWTH Aachen University Hospital, 52074 Aachen, Germany; fgremse@ukaachen.de (F.G.); srosenhain@ukaachen.de (S.R.); 6 Department of Neuroanatomy, RWTH Aachen University Hospital, 52074 Aachen, Germany; aslowik@ukaachen.de; 7 Department of Pathology, RWTH Aachen University Hospital, 52074 Aachen, Germany; mbienert@ukaachen.de (M.B.); sneuss-stein@ukaachen.de (S.N.); 8 Department of Orthopedic Surgery, Maastricht UMC+, 6229 HX Maastricht, The Netherlands; 9 Department of Trauma, University Hospital and University of Zurich, 9GGX+JM Zürich, Switzerland

**Keywords:** large bone defects, β-tricalcium phosphate, strontium, NF-κB, VEGFR-2, bioluminescence

## Abstract

It was hypothesized that strontium (Sr)-doped β-tricalcium phosphate (TCP)-based scaffolds have a positive effect on the regeneration of large bone defects (LBD). Readouts in our mice models were nuclear factor-kappa beta (NF-κB) activity and vascular endothelial growth factor receptor-2 (VEGFR-2) promoter activity during the healing process. A 2-mm critical-size femoral fracture was performed in transgenic NF-κB- and VEGFR-2-luciferase reporter mice. The fracture was filled with a 3D-printed β-TCP scaffold with or without Sr. A bioluminescence in-vivo imaging system was used to sequentially investigate NF-κB and VEGFR-2 expression for two months. After sacrifice, soft and osseous tissue formation in the fracture sites was histologically examined. NF-κB activity increased in the β-TCP + Sr group in the latter stage (day 40–60). VEGFR-2 activity increased in the + Sr group from days 0–15 but decreased and showed significantly less activity than the β-TCP and non-scaffold groups from days 40–60. The new bone formation and soft tissue formation in the + Sr group were significantly higher than in the β-TCP group, whereas the percentage of osseous tissue formation in the β-TCP group was significantly higher than in the β-TCP + Sr group. We analyzed longitudinal VEGFR-2 promoter activity and NF-κB activity profiles, as respective agents of angiogenesis and inflammation, during LBD healing. The extended inflammation phase and eventually more rapid resorption of scaffold caused by the addition of strontium accelerates temporary bridging of the fracture gaps. This finding has the potential to inform an improved treatment strategy for patients who suffer from osteoporosis.

## 1. Introduction

Bone possesses a high endogenous capacity for repair and regeneration. Nevertheless, delayed bone union and pseudarthrosis threaten the healing of large bone defects caused by tumor resection or trauma. This limited capacity for bone regeneration can often be observed in patients with oxidative stress-related diseases such as chronic inflammatory disease, type 2 diabetes mellitus, osteoporosis, alcohol abuse, or heavy smoking [1,2].

Early-phase fracture healing requires hematoma formation, which provides a static base for migrating inflammatory cells. The timely expiration of inflammation and early onset of revascularization periods are critical for soft tissue and callus formation. In contrast, a prolonged inflammation phase and circulatory impairment, separately or together, disturb the regular healing process [3,4]. Nuclear factor-kappa beta (NF-κB), an inducer of numerous proinflammatory cytokines and chemokines, is reportedly a key indicator in inflammation [5]. Impaired fracture healing is also associated with inadequate revascularization. Vascular endothelial growth factor (VEGF) is critical in neoangiogenesis at the fracture site. One previous study in animals has shown that blocking the VEGF receptors inhibits vascular in-growth, consequently delaying or disrupting the regenerative process, whereas sufficient VEGF promotes fracture healing [6]. In particular, VEGFR-2 is reported to be a good indicator during the bone-healing process [7]. However, there has been little discussion of the in-vivo longitudinal behavior of these inflammation and angiogenesis markers during the fracture-healing process. Bioluminescence in vivo imaging systems can provide real-time analysis of the various genes in order to address this gap [8,9].

Although the gold standard bone-grafting material is still an autologous graft, the attendant harvesting of bone material constitutes a secondary surgery. Common materials currently being studied for their potential as synthetic bone substitutes are collagen, hydroxyapatite (HA), calcium phosphate scaffolds (CaPs), calcium phosphate-types of cement, and glass ceramics [10,11]. Among these, β-tricalcium phosphate (TCP) is one of the most essential and most extensively investigated degradable scaffolds used in the repair of bony defects [12,13,14,15,16,17,18]. Recent studies have harnessed the biodegradation mechanisms, resorption, and fast bony adaption behavior in vivo of β-TCP scaffolds [19,20]. Many reports have focused on the effect of single–ion substitutions, such as by strontium (Sr), in the β-TCP. Strontium is well-known to enhance bone regeneration. Since the chemical properties of Sr and Ca are very similar, the Sr can be incorporated into the bone structure, identical to Ca, in the mineral phase [21]. Experimental and clinical studies refer to some beneficial effects of strontium ranelate in the turnover of abnormal (and, in particular, of osteoporotic) bone, including increased osteogenesis and bone formation, as well as reduced fracture incidence, even when administered systemically by oral application [22,23]. Nevertheless, the oral application of strontium ranelate increases the risk of deep venous thromboembolism due to systemic distribution and, therefore, should be avoided in patients with the increased risk of stroke and ischemic cardiac disease [24]. The exact mechanism behind the action of strontium still needs to be elucidated [25,26].

Therefore, we hypothesized that (a) the longitudinal analysis of inflammation and angiogenesis during the bone-healing process in transgenic mice would be a beneficial alternative to endpoint-fracture models, as it would reduce the number of animals involved, (b) the novel calcium phosphate-based scaffold would enhance the healing process in critical-size bone defects, and c) the introduction of Sr into this scaffold would elevate bone formation and accelerate gap-bridging in our critical-size bone fracture model. The aim of this study was to investigate the potential effects of novel β-TCP scaffolds (with or without Sr) on bone formation in critical-size femur defects in mice and to longitudinally evaluate inflammation and angiogenesis during the healing process with transgenic NF-κB- and VEGFR-2-luciferase reporter mice.

## 2. Results

### 2.1. Longitudinal Monitoring of NF-κB Activity

**Figure 1:**
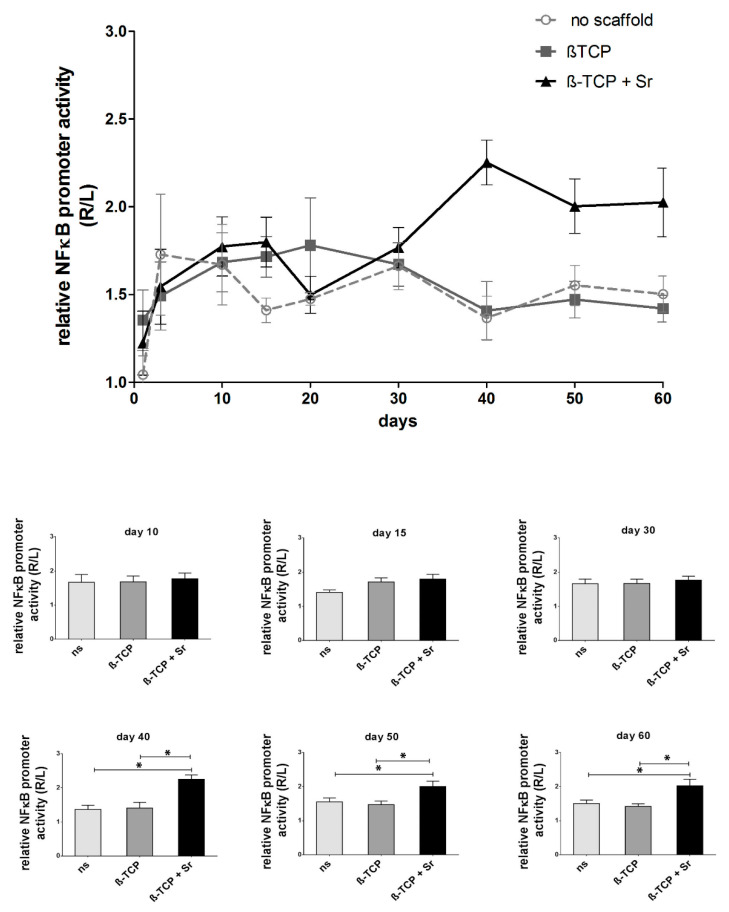
Longitudinal tracking of nuclear factor-kappa beta (NF-ĸB) activity during fracture healing in transgenic mice. NF-κB promoter activity in the fracture area was measured as a luminescence signal and appears as average radiance in the region of interest (ROI), as detected on a Xenogen imaging system. Sr increased inflammation by means of NF-κB activity in the late healing stage. The non-scaffold group is a nonunion group (n ≥ 4; *p* ≤ 0.05). βTCP: β-tricalcium phosphate, *p* value is given as *p* ≤ 0.05. Asterisks indicate statistical differences within the groups.

NF-κB promoter activity in the fracture area was measured as a luminescence signal and detected on a Xenogen imaging system. The longitudinal data (Figure 1) reveal increased NF-κB activity in all groups up to the tenth day (with fold changes of 1.67 ± 0.22 in the control group and 1.68 ± 0.17 and 1.77 ± 0.19 in the β-TCP and β-TCP + Sr groups, respectively). NF-κB promoter activity decreases continuously in the control group (1.47 ± 0.04 at day 20, 1.66 ± 0.13 at day 30, 1.36 ± 0.13 at day 40, 1.55 ± 0.11 at day 50, and 1.50 ± 0.10 at day 60; n = 4). In the group with β-TCP scaffolds, NF-κB promoter activity exhibits its highest peak at day 20 (1.78 ± 0.27) and decreases continuously down to the level of the control group (1.67 ± 0.12 at day 30, 1.41 ± 0.17 at day 40, 1.47 ± 0.10 at day 50, and 1.42 ± 0.08 at day 60; n = 5). In contrast, the + Sr group shows the least promoter activity at day 20 (1.50 ± 0.26), though it increases again significantly in the latter stage of healing, peaking after 40 days (1.77 ± 0.11 at day 30, 2.25 ± 0.13 at day 40, 2.00 ± 0.16 at day 50, and 2.02 ± 0.19 at day 60; n ≥ 6; *p* ≤ 0.05).

### 2.2. Longitudinal Monitoring of VEGFR-2 Activity

VEGFR-2 promoter activity in the fracture area correlates with measured luminescence signals (appearing as average radiance) in the region of interest (ROI), as detected on a Xenogen imaging System.

**Figure 2:**
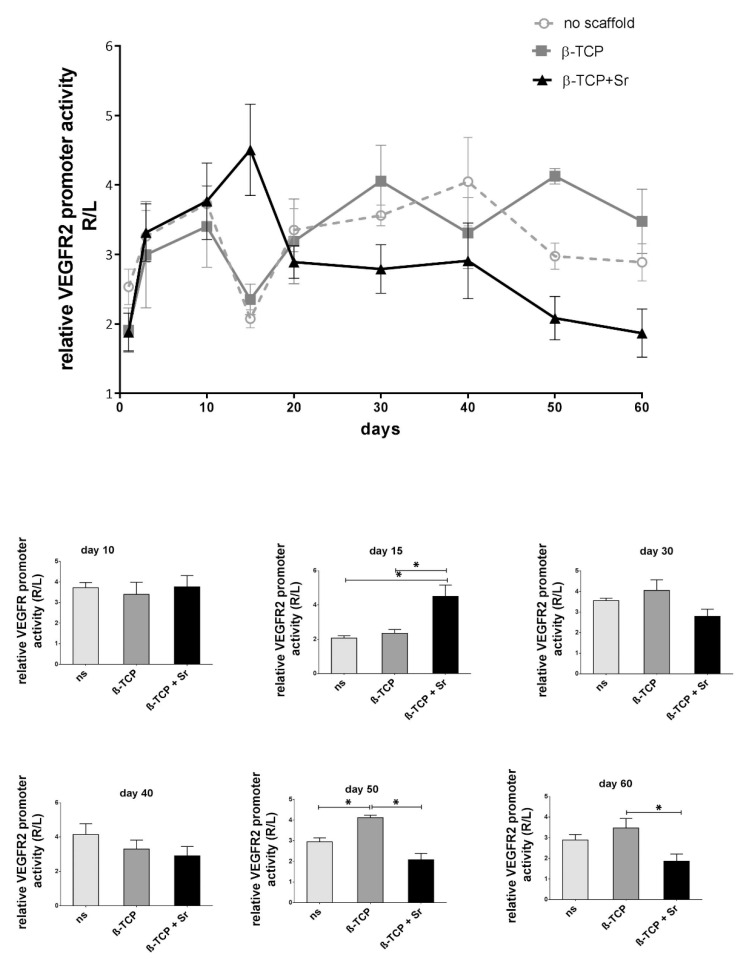
Longitudinal tracking of vascular endothelial growth factor receptor-2 (VEGFR-2) activity during fracture healing in transgenic mice. VEGFR-2 promoter activity in the fracture area was measured as a luminescence signal and appears as the average radiance in the region of interest (ROI), as detected on a Xenogen imaging system. We observed the first peaks of luciferase activity on day 10 (in the early angiogenesis period) in all groups. While the level of VEGFR-2 activity increases in the Sr-doped β-TCP (β-TCP + Sr) group on day 15, luciferase activity starts to decrease in this group and eventually shows significantly less activity here than in the other two groups in the second half (n ≥ 4; *p* value is given as *p* ≤ 0.05. Asterisks indicate statistical differences within the groups).

The longitudinal data (Figure 2) reveal an increase in VEGFR-2 activity in all groups up to the tenth day (3.73 ± 0.25 in the control group and 3.40 ± 0.59 and 3.76 ± 0.55 in the β-TCP and β-TCP + Sr groups, respectively). VEGFR-2 promoter activity decreases both in the control and β-TCP groups at day 15 (2.07 ± 0.13 and 2.35 ± 0.22, respectively) but steadily increases in the latter phase of the fracture healing period (3.55 ± 0.31 at day 20, 3.56 ± 0.11 at day 30, 4.15 ± 0.63 at day 40, 2.95 ± 0.19 at day 50, and 2.89 ±0.27 at day 60 in the control group; n = 4, and 3.19 ± 0.61 at day 20, 4.05 ± 0.52 at day 30, 3.31 ± 0.52 at day 40, 4.12 ± 0.11 at day 50, and 3.47 ± 0.65 at day 60 in the β-TCP group; n = 4). The + Sr group shows the highest peak in promoter activity at day 15 (4.50 ± 0.66), but unlike in the other groups, promoter activity in the + Sr group decreases continuously and remains stably below the level of the β-TCP group in the second half of the healing period (2.89 ± 0.23 at day 20, 2.80 ± 0.35 at day 30, 2.91 ± 0.54 at day 40, 2.08 ± 0.31 at day 50, and 1.87 ± 0.35 at day 60; n = 4; *p* ≤ 0.05).

### 2.3. Histological Analysis of Tissue Formation

**Figure 3:**
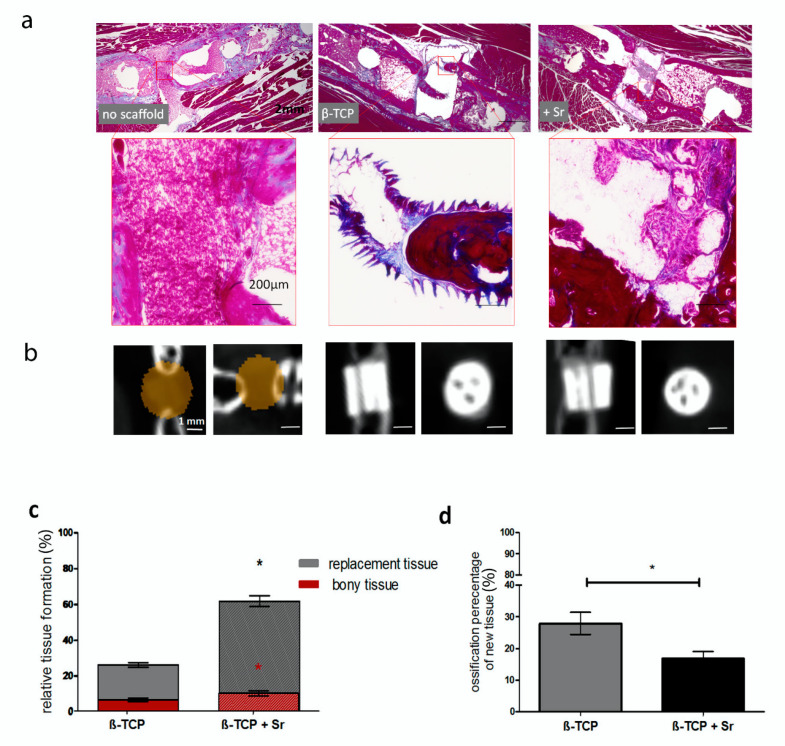
Histological analysis of callus formation. In both the β-TCP and β-TCP + Sr groups, the connections between newly formed tissue within the scaffolds’ integrated canals was clearly visible. In the non-scaffold control group, a connective tissue formation was observed (**a**,**b**). (**b**) Micro-CT analysis shows coronal (i) and transversal (ii) sections of a scaffold after two months. The brown cylinder marked with an asterisk represents the soft tissue in fracture gaps in the control group. The channels in both β-TCP and β-TCP + Sr groups are filled with or replaced by soft and bony tissue (gray). (**c**) The y-axis shows the percentages of newly formed (gray) and bony (red) tissues in relation to the scaffold area. Our histological analysis showed that much more tissue had been built in the β-TCP + Sr group than in the β-TCP group. The y-axis shows the percentage of osseous tissue (red) in relation to the scaffold area (gray). Bone formation in the β-TCP + Sr group was significantly higher than in the β-TCP group (red asterisk). (**d**) In contrast, the percentage of osseous tissue among all newly formed tissue was much higher in the β-TCP scaffold group than in the β-TCP + Sr scaffold group (n ≥ 4; *p* ≤ 0.05. Red asterisks indicate statistical differences of relative bony tissue formation and black asterisks indicate statistical differences of relative tissue replacement within the groups).

Our histological examination revealed that osseous tissue had formed after two months but only where scaffolds were employed. In the control group, with no scaffolds, the entire defective area was filled with soft, connective tissue (Figure 3a). Our µCT analysis verified the lack of bony tissue between bone gaps in the control group, whereas the channels had been filled in or replaced with bony tissue in other groups (Figure 3b).

In both the β-TCP and β-TCP + Sr groups, the newly formed tissue within the scaffold area was visibly connected to the ends of the fracture. The + Sr group showed significantly higher tissue regeneration (61.93% ± 3.04%) than the β-TCP group (26.41% ± 1.31%) (Figure 3c).

We also determined the percentage of ossified tissue and amount of ossified tissue in proportion to all the newly formed tissue. The percentage of bone formation in the β-TCP + Sr group (10.13% ± 1.4%) was significantly higher than in the β-TCP group (6.53% ± 0.99%) (red column). The relative amount of ossified tissue to all newly formed tissue was higher in the β-TCP group than in the β-TCP + Sr group. In the β-TCP group, 27.92% ± 3.45% of all tissue was ossified, whereas only 16.80% ± 2.30% of the tissue in the β-TCP + Sr group could be identified as ossified (Figure 3d).

### 2.4. Relative Protein Expression of Osterix (Osx) in Scaffolds

**Figure 4:**
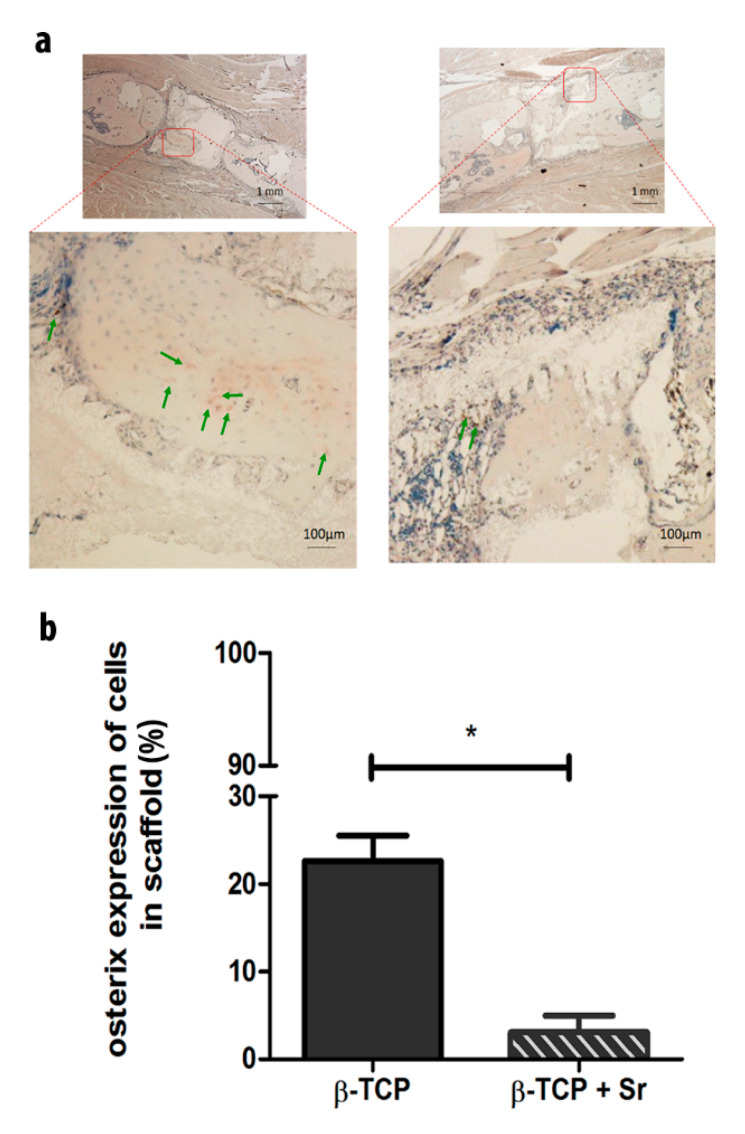
Relative protein expression of Osterix in the scaffold. (**a**) The images represent sections that have been immunohistochemically stained against Osterix (Osx) in the vicinity of newly formed tissue in the scaffold (red square) in both the β-TCP (left) and β-TCP + Sr (right) groups. Green arrows indicate Osx-positive cells shown up by red staining. (**b**) The ratio of Osx-positive to total cells in the β-TCP group was significantly higher than in the β-TCP + Sr group (n ≥ 3; *p* value is given as *p* ≤ 0.05. Asterisks indicate statistical differences within the groups).

To verify the examined Osx protein expression data in newly formed tissues in the scaffolds, we performed an immunohistochemistry analysis (Figure 4a) and calculated the ratio of Osx-positive cells to total cells (Figure 4b). The percentage of Osx-positive cells in the β-TCP group (22.60% ± 2.88%) was significantly higher than in the β-TCP + Sr group (3.08% ± 1.90%).

### 2.5. Quantification of Bone-Marker Levels in Serum

Using a Luminex^®^ technology, the protein levels of fibroblast growth factor 23 (FGF-23), osteocalcin (OC), osteoprotegerin (OPG), Dickkopf-related protein 1 (DKK), insulin, and leptin were quantified in the animals both before and two months after the operation.

**Figure 5:**
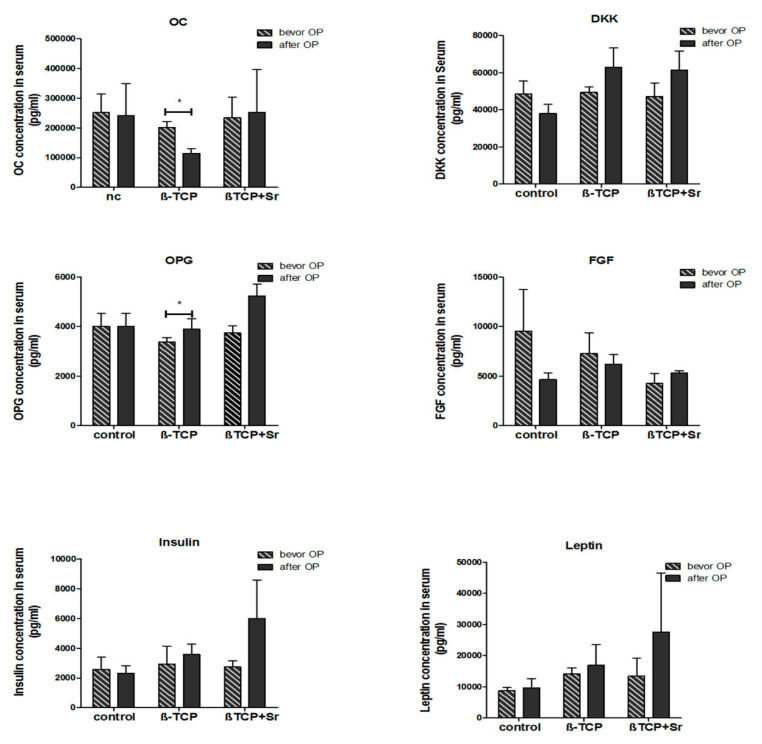
Quantification of growth factors in the serum. Levels of fibroblast growth factor (FGF), osteocalcin (OC), osteoprotegerin (OPG), Dickkopf-related protein 1 (DKK), insulin, and leptin were quantified using the Luminex system in the serum. These markers were compared among all three (control, β-TCP, and β-TCP + Sr) groups both before, as well as two months after, surgery (n = 4). * *p* ≤ 0.05 indicates significance.

The results showed a significant reduction of osteocalcin levels in the β-TCP group (201,640 ± 20,183 pg/mL vs. 114,419 ± 15,713 pg/mL; n = 4; *p* ≤ 0.05) and a significant increase in osteoprotegerin levels in the β-TCP + Sr group (3736 ± 200.5 pg/mL vs. 5232 ± 338.9 pg/mL; n = 4; *p* ≤ 0.05) during the healing process. No significant differences in the protein levels of FGF, DKK, insulin, and leptin were to be observed among the groups, neither before nor two months after surgery (Figure 5).

## 3. Discussion

### 3.1. Use of Transgenic Mice

The use of animal models has made it possible to study fracture healing from various perspectives, such as histology, biochemistry, and biomechanics, and has hence been an essential tool for the development of improved therapies to bone regeneration [27].

Mice fracture models are economical and widely used to analyze the various molecular mechanisms of bone regeneration and the healing process. The longitudinal in-vivo techniques allow a further reduction of the number of animals and arrange to get the interim data about the animal. Most published data are based on µCT, histochemistry, and immunohistochemistry. µCT is the gold standard method for the evaluation of mineralized tissue. Using of µCT by analysis of fractures, which are stabilized by external fixators, are an elaborate procedure to the presence of screws and metal parts used to stabilize the fracture [28]. Additionally, the mineralization of a fracture is proceeded by assuming soft fiber and cartilage tissue of the callus. µCT analysis cannot deliver precise data about the process of the initiate inflammation period or vascularization and soft callus formation in the early phase of fracture healing [29,30]. The local inflammatory process could be illustrated, so far, mostly in two-week intervals after euthanizing the animal using histological and immunohistological staining. Haffner-Luntzer et al. established an MRI-compatible osteosynthesis device to evaluate the callus formation in mice femurs in vivo [31]. The MRI technique is not a sufficient longitudinal method for the investigation of a critical-size fracture due to the application of a metallic part of the fixator. Bioluminescence in-vivo imaging systems allow real-time analysis of various genes [8,9] and is used in our study.

### 3.2. Longitudinal NF-κB Activity during Fracture Healing

The present study, which investigates the longitudinal course of NF-κB activity during the fracture-healing process, is the first of its kind. Our results, which show increased NF-κB activity in the early phase of fracture healing in all groups, point to the intervent, intervened inflammatory response, which we know is associated with bone defects. Three weeks after fracture, NF-κB activity in the β-TCP group is tendentially higher than it is in the β-TCP group, with strontium scaffolds (+ Sr), and this would indicate that strontium has anti-inflammatory effects during the early phase of fracture healing. Previous studies have reported that strontium can have immunomodulatory properties [25]. Lourenco et al. found that fewer inflammatory cells were recruited to a strontium implant site than to that of a sham operation in rodents [25]. Similar to our study, they obtained their results within around two weeks of implantation. However, the specific amount of strontium in the scaffolds can affect the release of Sr^2+^ [32].

On the other hand, NF-κB activity tended to decrease in the latter phase of fracture healing in both the non-scaffolded as well as the β-TCP-scaffolded group, but in the strontium (+ Sr) group, by contrast, NF-κB activity shows a significant increase beginning around day 40 after the fracture. The immunomodulatory properties of Sr is discussed in some studies [25,33], but as far as we know, such an anti-inflammatory effect of strontium has never been reported in a study featuring longitudinal monitoring. Acute inflammation is a crucial biological process for tissue homeostasis in the early phase of fracture healing. Successful clearance of inflammatory stimulation due to anti-inflammatory and reparative cytokines generally resolves the inflammatory situation and re-establishes tissue homeostasis [34,35,36,37]. Conversely, persistent proinflammation can result in chronic inflammation and impaired fracture healing [38,39,40]. Diseases characterized by chronic inflammation (such as osteoarthritis or rheumatoid arthritis) increase the risk of nonunion after fracture [41,42]. Our study indicates there is a possibility that strontium may lead to impaired fracture healing in a chronic inflammation environment.

### 3.3. Longitudinal VEGFR-2 Activity during Fracture Healing

In the present study, VEGFR-2 activity increased immediately after the fracture in all groups, which suggests neovascularization in the bone defect in the early phase of bone healing. Revascularization after the injury acts as a critical process for soft callus formation during regeneration [43]. Two weeks after the fracture, activity decreased in both the β-TCP and non-scaffolded groups. This stands in contrast with an increase in VEGFR-2 activity in the strontium (+ Sr) group two weeks after the fracture, which suggests that strontium may promote vascular formation and tissue repair by activating VEGF in the reparative phase of fracture healing. Although VEGFR-2 activity in the strontium group declined in the latter stage, strontium may have a positive effect on tissue formation toward fracture healing in the long run.

On the other hand, VEGFR-2 activity exhibited sequentially higher values in the β-TCP group than in the non-scaffolded group. The interdependency of the respective time cascades of inflammation, angiogenesis, and tissue regeneration is described by Schmidt-Bleek et al. [3]. Our current longitudinal results indicate the β-TCP scaffold had a positive effect on fracture healing because of the associated continuous activation of VEGFR-2.

### 3.4. Bone and Tissue Regeneration

Our histopathological findings, which we obtained two months after the fracture, reflect significantly higher relative tissue formations in the β-TCP + Sr group than in the non-strontium β-TCP group.

VEGF positively influences the soft callus formation through neovascularization during the fracture-healing process [44,45]. Thus, a further increase in VEGFR-2 activity two weeks after the fracture might have led to higher relative tissue formation in the strontium (+ Sr) group, even two months after the fracture. The level of OPG in the serum in the strontium group was likewise significantly higher two months after surgery than prior to it, and serum levels of the OC remained constant in the + Sr group after surgery, though they decreased significantly in the other groups. On the other hand, our histopathology showed a significant decrease in the bone percentage of new tissue in the β-TCP + Sr group compared with the non-strontium β-TCP group. Clinically, diseases associated with chronic inflammation—including alcoholism, (morbid) obesity, osteoarthritis, rheumatoid arthritis, and type II diabetes—increase the risk of nonunion [41,42]. Our present findings therefore suggest that chronic inflammation associated with NF-κB activation in the latter phase of fracture healing can disturb bone repair.

The short initial inflammatory phase and higher percentage of bony tissue relative to the + Sr group suggests that our β-TCP group-type characteristics have a potential beneficial application in treating critical-size fractures.

Nevertheless, the + Sr group exhibited greater tissue formation (albeit with a lower percentage of bone), and these soft-and-bony tissue bridges filled the fracture gaps faster than was the case in the β-TCP group. This effect may prove advantageous for fracture healing in patients who exhibit lowered bone stability, such as those with osteoporosis.

Osterix (Osx) is an essential transcription factor for osteoblast differentiation; bone cannot form without it. Takebe et al. was able to observe Osx-positive cells on the surface of a newly formed bone matrix seven days after the fracture [46]. We saw very few Osx-positive cells, particularly in the + Sr and non-scaffold groups. (Non-scaffold group data are not shown.) This may be attributable to the fact that we analyzed Osx expression in the latter phase of fracture healing. The effect of Sr on Osx expression is not sufficiently understood, and the existing data on Osx expression during Sr treatment is controversial. Peng et al. found no significant changes in Osx gene expression in either their Sr or control groups [47]. Liu et al., on the other hand, reported a dose-dependent increase in Osx-mRNA in defective bone tissues implanted with 0%, 5%, or 10% Sr-CaS for eight weeks [48].

### 3.5. Feasible Size of Femur Defect in Mice

For the present study, we used a 3D-printed scaffold with a diameter of 2 mm and a height of 1.7 mm for a 2-mm bone defect in mice femur diaphyses. Accordingly, no sufficient bone or soft tissues were obtained in the fracture gap without scaffolds. Additionally, previous studies have found substantial bone formation in scaffolds inserted into a 2.3-mm bone defect in mice femurs [49,50]. Our critical-size femur defect models in mice would therefore be useful for further studies of the role of other scaffolds in fracture healing.

### 3.6. Strontium-Doped β-TCP Scaffold

Strontium salts as strontium citrate, strontium chloride, or strontium ranelate have shown to overcome the bone loss in the in-vivo and in-vitro experiments [51,52]. Strontium citrate and chloride are supplements, and their effectiveness is not sufficiently investigated so far. Oral administration of strontium ranelate for the treatment of osteoporosis was approved for a long time [53]. Nevertheless, the oral application of strontium ranelate increases the risk of deep venous thromboembolism due to systemic distribution and, therefore, should be avoided in patients with an increased risk of stroke and ischemic cardiac disease [24]. To explore the positive effects of strontium, many reports have focused on the local application of Sr as single–ion substitutions in the scaffolds [54,55,56,57]. We used Sr-doped β-TCP in this study. The scaffolds were provided with channels inside. A diameter of 100 μm or more has been shown to support new bone formation [58,59,60]. The used diameter of 300 µm in this study mimics the trabecular structure of bone. Despite macroscopically identical structures produced by the same molds, there was microscopical differences between the β-TCP scaffold and β-TCP + Sr scaffold due to the average pore diameter, porosity, and total pore surface. Barba et al. have shown that pore clustering and architecture play a critical role in osteoinductivity of the scaffold. They observed that the sintered and biomimetic frameworks showed different bone formation patterns [61]. In-vitro studies have shown that the nanostructure of osteoinductive materials influence the differentiation of mesenchymal stem cells and upregulation of osteogenic genes without adding osteogenic factors [62]. The existing of fine differences in the microstructures of the used scaffolds in our study may have influenced the new bone formation.

### 3.7. Limitations

The main limitation of this study is that the skin incision of subcutaneous tissue in longitudinal monitoring can affect both NF-κB and VEGFR-2 activity. However, this method enables us to sequentially evaluate both NF-κB and VEGFR-2 activities in the same mice and to reduce the number of animal subjects.

A general limitation of longitudinal analyses of critical-size fracture healing, in which fractures are stabilized by either an external or an internal fixator, is the degradation of µCT quality. The β-TCP scaffold we used in the present study caused some degree of artifact in the µCT. The background signal impairs the analysis of the new bone formations.

Our results represent the healing process we observed over an eight-week period, but the healing process was not complete when we concluded our observations. An analysis of longer-term outcomes would be needed to compare the biomechanical properties of replaced tissue, as well as the bony tissue quality, in either group (with and without strontium).

## 4. Materials and Methods

### 4.1. Scaffold Manufacturing

**Figure 6:**
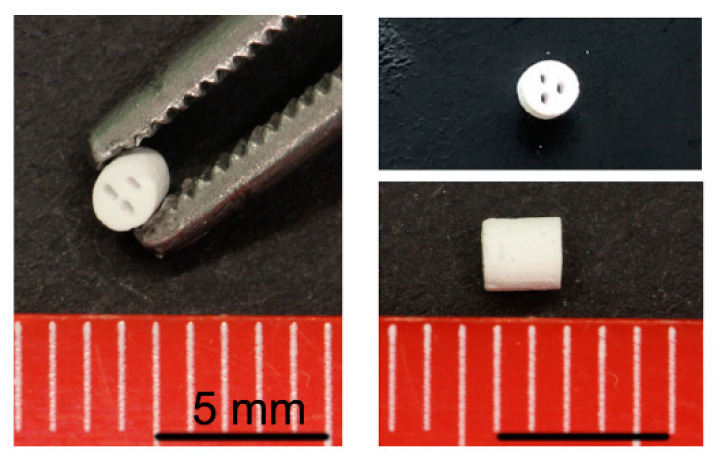
Macroscopic imaging of β-TCP scaffold.

**Figure 7:**
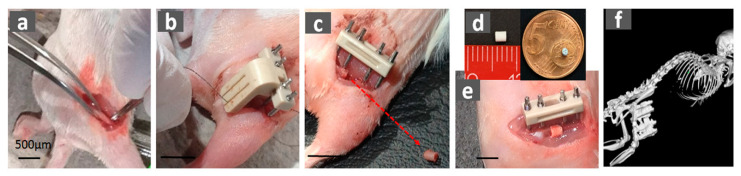
Implantation of novel β-tricalcium phosphate scaffolds in critical-size fractures. A longitudinal incision along the femur was performed (**a**). Then the Mouse ExFix (RISystem) was positioned on the femur. Subsequently, the bone was stabilized by an external fixator using four screws (**b**). Using the two grooves in the saw guide, a defined fracture gap of 2 mm was created with a Gigli saw (**c**). The wound was washed with Ringer’s solution, and the scaffold (**d**) was implanted into the fracture gap (**e**). µCT evaluates the position of the scaffold and external fixator (**f**).

The scaffolds used in this study were synthesized in accordance with Lindner et al. [12]. Strontium-doped calcium phosphate suspensions with high solid contents were prepared using organic liquefier, binder, and anti-foaming agents. The suspensions were cast into 3D-printed wax models for scaffold production. The filled wax models were fired at 1200 °C for 3 h. Scaffolds manufactured by slip-casting in 3D-printed wax molds were successfully adapted to fit into a critical-size defect mouse model (Figure 6 and Figure 7d,e). The scaffolds were downscaled to a diameter of 2 mm and a height of 1.7 mm. The β-TCP scaffold used in this study had a porosity of 24.5 vol%. The total pore surface area was 0.3 m^2^/g, with an average pore diameter of 2.0 μm. The β-TCP + Sr scaffolds had a porosity of 22.1 vol%. The total pore surface was 0.4 m^2^/g, and the average pore diameter was 1.5 μm. The scaffolds exhibited a compression strength of 9.4 ± 2.1 MPa. The solubility of strontium in PBS was 65 ng/mm^2^ after 21 days. We also characterized the cytocompatibility of both scaffolds [58]. In addition to the micropores, having a macro-porosity of more than 100 µm is evidence to mimic the trabecular bone infrastructure and guarantee the cell migration and vascularization in the scaffold [59]. Therefore, we used three macro-canals to warrant the trabecular building and capillary formation.

### 4.2. Transgenic Animals

For in-vivo imaging purposes, transgenic reporter mice have been genetically engineered with a luciferase gene cloned to transcriptional promoters that are responsible for the expression of VEGFR-2 or NF-κB.

For this study, we used twelve luciferase transgenic mice of the strain (BALB/C-Tg (NF-κB-RE-luc)-Xen and twelve luciferase transgenic mice of the strain FVB/N-Tg (Vegfr-2-luc)-Xen), all between 19–21 weeks of age.

All mice were kept in suitable mouse cages and acclimated for one week prior to the start of the study. Mice were exposed to a 12-h light-and-dark cycle via artificial illumination (light period: 7 a.m. to 7 p.m.) and kept at a controlled room temperature of 22 ± 2 °C in specific pathogen-free conditions. Food pellets and water were available ad libitum. Autoclaved wood chippings were used as bedding material. All procedures were performed with the approval of the appropriate governmental institution (Landesamt für Natur-, Umwelt- und Verbraucherschutz, LANUV NRW, Bonn, Germany, reference number: AZ 84-02.04.2012.A029).

### 4.3. Critical-Size Fracture Model (Large Bone Defect)

Before surgery, the lower backsides of the mice were shaved and disinfected. Anesthesia was induced with intraperitoneal administration of a mixture of xylazine (8 mg/kg animal weight) and ketamine (100 mg/kg animal weight). After administration of the narcotic agent, surgical tolerance was tested by toe pinch and whiskers’ reflexes to ensure deep anesthesia. Bepanthen eye cream was applied to avoid desiccation, and the animals were placed on a warming mat for the duration of the surgery.

A longitudinal incision was performed along the femur. The fascia and muscles were removed to expose the length of the femur (Figure 7a). Then a Mouse Ex-Fix was attached to a saw-guide fixator positioned on the prepared femur in an anterolateral direction parallel to the longitudinal axis of the bone. The bone was subsequently stabilized by an external fixator using four screws (RISystem, Davos, Switzerland) [28].

Using the two grooves in the saw guide (Figure 7b), a defined fracture gap of 2 mm was created using a 0.22-mm Gigli saw (Figure 7c; RISystem, Davos, Switzerland) in the middle of the femur. The wound was washed with ringer solution, and the scaffold was implanted into the fracture gap via press-fitting (Figure 7d,e). One group received the osteotomy but no scaffold. We were also able to use X-ray and micro-computed tomography (µCT) to monitor how well the scaffold position was maintained (Figure 7f,g).

The muscular layer and fascia were sutured first; the skin was sutured later. After surgery, the animals were closely watched until they awoke from anesthesia. They were exposed to infrared light to stave off hypothermia. Additionally, a dose of buprenorphine (0.1 mg/kg body weight) was injected subcutaneously to ensure proper analgesia. Buprenorphine application continued post-surgery twice a day for 7 days.

In the first group, an external fixator was attached and an osteotomy performed without implanting a scaffold. The second group consisted of animals that had been implanted with a β-TCP scaffold. The third group contained animals implanted with a β-TCP + Sr scaffold.

### 4.4. In-Vivo Luminescent Imaging

**Figure 8:**
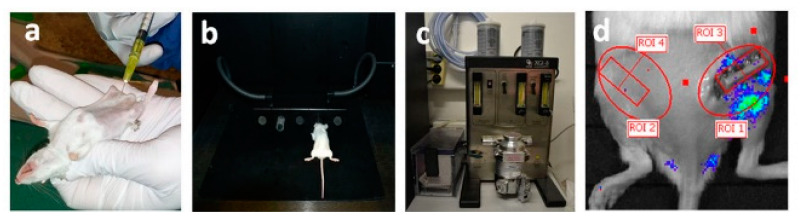
Longitudinal monitoring of ongoing angiogenesis and inflammatory processes by detecting bioluminescence signals in vivo. The expression levels of VEGFR-2 and the activity of NF-κB (representing either angiogenesis or inflammation) were monitored noninvasively, in longitudinal fashion, using the Xenogen imaging system (IVIS Lumina Imaging System 100) (Caliper Life Sciences, Hopkinton, MA, USA). Luciferase activity was visualized after intraperitoneal injection of 200µL of D-luciferin (150 mg/kg) (**a**). Mice were placed in the chamber (IVIS Lumina, Caliper Life Sciences, Hopkinton, MA, USA) (**b**) under continuous anesthesia (2.5% isoflurane and 2 L/min oxygen flow by inhalation (**c**) 10 min before imaging (to allow D-luciferin distribution). To quantify the luminescence, elliptical regions of interest (ROIs) were placed on the right and left limb (ROI 1 and ROI 2) (**d**). At each time point, ROI luminescence was measured as the average radiance (photons per second per square centimeter per steradian: p/s/cm^2^/sr) for all animals. To reduce the false negative effect of the fixator plate, signals from the second square ROI in the same volume as the fixator plate (ROI 3 and ROI 4) were subtracted from both the right and left elliptical regions (**d**).

The mice received an intraperitoneal injection of 200 µL of D-luciferin (150 mg/kg) 10 min before imaging to allow time for D-luciferin distribution. Luciferase activity was visualized afterwards. The D-luciferin solution (Synchem OHG, Felsberg/Altenburg, Germany) was prepared as described by Fet et al. [60]. The mice were placed in the chamber (IVIS Lumina, Caliper Life Sciences, Hopkinton, MA, USA) under continuous anesthesia (2.5% isoflurane and 2 L/min oxygen flow by inhalation). Whole-body NF-κB or VEGFR-2 activity-related luminescence was then imaged on an in-vivo imaging system. Bioluminescence measurements were taken before the surgical intervention and then after it once a week [6]. Measurement parameters were set to 10 min integration time and the field of view to ten, with medium sensitivity of the Charge Coupled Devices (CCD) camera (binning = 4) (Figure 8).

To quantify luminescence, elliptical regions of interest (ROI) were placed on the right and left limbs. The ROIs were selected to encompass the majority of the respective variances in NF-κB or VEGFR-2 activity, as observed in the imaging dataset. At the time of each observation, ROI luminescence was measured as the average radiance (in photons per second per square centimeter per steradian: p/s/cm^2^/sr) for all animals. To reduce any false negative effects from the fixator plate, we subtracted the second square ROI in the same volume as the fixator plate from both the right and the left elliptical ROIs (Figure 8d). Luminescence data for each animal and each point in time was normalized to luminescence values from the contralateral side of the same animal.

### 4.5. Micro-CT Imaging

The mice were scanned in a small-animal dual-source µCT (TomoScope Duo, CT-Imaging, Erlangen, Germany). They were anesthetized during imaging by an application of isoflurane (2% isoflurane in air; flow rate: 1 L/min). The sources of the flat-panel µCT were operated at a voltage of 65 kV and a current of 0.5 mA. A 90-s, full-rotation scan acquired 720 projections at a resolution of 1032 × 1012 pixels [61].

Reconstructions were performed with voxel sizes of 35 µm using a Feldkamp-type algorithm. The volumetric images were visualized and analyzed using the Imalytics Preclinical software [62].

### 4.6. Histological Staining

The femur samples were prepared and placed into methacarn solution for fixation lasting a total of 24 h. Subsequently, the legs were decalcified in a 10% EDTA buffer for 21 days; then, they were washed under running tap water and dehydrated in an ascending ethanol series (from 70% to 100% ethanol). Afterwards, tissues were first placed in xylene and, then, in 56 °C preheated paraffin wax (for paraffin infiltration). Tissues were then embedded in paraffin and left to solidify at room temperature (RT). The paraffin block was cut into 4.5-µm-thick sagittal sections using a microtome. The sections were left to dry and kept at RT to await further processing.

### 4.7. AZAN Staining

**Figure 9:**
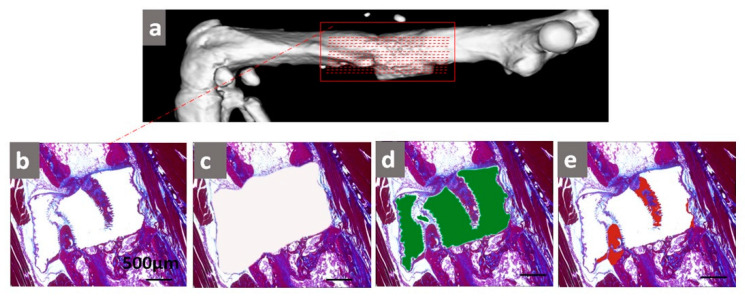
Histological analysis of the fracture site. Micro-CT image of the femur after two months (**a**). The red line represents the histological serial section of the femur (**a**). The critical-size defect was visualized using azan staining. Regenerated tissue formation was stained in the channel holes of the scaffold (**b**). The scaffolded area (white, **c**), tissue-free area (green, **d**), and bone formation (red, **e**) were quantified and used to calculate the amounts of regenerated tissues and bone for each group after two months.

Approximately one out of every fifteen serial sections of femur were used for azan staining to visualize the formation of both soft and hard tissue (Figure 9). Azan staining was carried out in accordance with the standard protocols described by Mulisch and Welsch et al. [60,63]. Acocarmin G and Anilinblau-Goldorange are used for a trichrome stain to mark different tissues histologically. Azan stains muscle fibers and osseous tissues red and cartilage/bone matrix and fibrous tissues blue.

The area around the scaffold, the tissue-free area, and the bone area were examined using a microscopical approach (Keyence BZ-9000; Keyence, Osaka, Japan). To measure the area of newly formed tissue, the tissue-free area was subtracted from the total area of the scaffold (Figure 9). Relative tissue formation and relative bone formation were determined by the following equations:

(1) $$\mathrm{relative}\;\mathrm{tissue}\;\mathrm{formation}\;\left(\%\right)=\left(\frac{new\;tissue\;area}{total\;scaffold\;area}\right)\;\cdot \;100$$

(2) $$\mathrm{relative}\;\mathrm{bone}\;\mathrm{formation}\;\left(\%\right)=\left(\frac{new\;bone\;area}{total\;scaffold\;area}\right)\;\cdot \;100$$

Average values were used for data presentation.

### 4.8. Immunohistochemical Staining Analysis

Routine immunohistochemical procedures were performed on various slides from each group. The deparaffinized sections were soaked in 3% hydrogen peroxide (H_2_O_2_) in methanol at room temperature for 15 min to inhibit endogenous peroxidase activity. The sections were then washed 3 times with Tris-buffered saline (TBS) and blocked with swine serum (Agilent DAKO, Santa Clara, CA, USA, Cat# x0901; 1:20 in TBS). Afterwards, sections were incubated for 24 h at 4 °C with primary antibodies against Osterix (Osx, Santa Cruz Biotechnology, Heidelberg, Germany; Cat# Sc22536-R; 1:250 in 1.5% BSA in TBS). The primary antibody was omitted in the negative control experiments. After being washed for 3 × 5 min in TBS, the sections were incubated with biotinylated polyclonal swine anti-rabbit immunoglobulins (Agilent DAKO, Santa Clara, CA, USA, Cat#E0353; 1:400) as secondary antibodies for 30 min. The sections were then visualized with an AEC-Kit (Invitrogen Corporation, Carlsbad, CA, USA) for 5 min. They were then counterstained with hematoxylin. Images of the scaffold area were taken using a Keyence BZ-9000 microscope (Keyence, Osaka, Japan) with a 20× objective lens. The protein expression of Osx in the scaffold area was determined by the relative value of positive-stained cells to total cells in the new tissue area in at least three different regions of the same section as ascertained by two examiners who were blinded to the project. The mean of all values per section was then calculated.

### 4.9. Quantification of Serum Protein Level

The concentrations of osteocalcin, osteoprotegerin, and fibroblast growth factor 23 (FGF-23) were analyzed using a Luminex multiplex immunobead assay (Luminex Corp., Austin, TX, USA) based on xMAP technology. The BioPlex kit (Bio-Rad Laboratories, Hercules, CA, USA) was performed strictly as described in the manufacturer’s guidelines. Two-hundred microliters of whole blood was collected from the retrobulbar area and left undisturbed to clot. Serum was separated by centrifugation at 18,000× *g* for 1 min and then stored at −80 °C. The serum sample was diluted 1:4 with the serum dilution included in the kit.

### 4.10. Statistical Methods

Prior to statistical analysis, residuals were tested for normal distribution with the Shapiro-Wilk normality test and for variance homogeneity with the Bartlett’s test. If one of the aforementioned tests were significant, the data were transformed, and residual normal distribution and variance homogeneity were then re-evaluated. Intergroup differences were tested by one-way ANOVA (except for the longitudinal data) or two-way ANOVA (repeated measure, using Greenhouse–Geisser corrections), followed by Tukey’s post-hoc test. All statistical calculations and data presentations were performed using GraphPad Prism, version 8.2.0 for Mac, GraphPad Software, San Diego, CA, USA, www.graphpad.com. Data are given as a mean ± SEM. The level of significance was set at *p* ≤ 0.05 (*).

## 5. Conclusions

We present first-time profiles of the longitudinal activities of VEGFR-2 promoter and NF-κB—respective agents of angiogenesis and inflammation—during critical-size bone-fracture healing.

We found high percentages of osseous tissue in our β-TCP group, which could be the result of high VEGFR-2-promoter activity in late-phase fracture healing.

The addition of strontium to calcium phosphate-based scaffolds influenced the inflammatory response at various stages of the healing process and may have affected bone regeneration. An extended inflammation phase caused by the addition of strontium accelerates tissue formation and, thus, the bridging of fracture gaps, which may aid in the treatment of patients suffering from osteoporosis.

The precise impact of strontium-doped β-TCP in osteoporotic large-bone defects bears further study.

## Abbreviations

β-TCP	β-tricalcium phosphate
CCD	Charge Coupled Devices
FGF	Fibroblast growth factor
IGF	Insulin-like growth factor
IL	Interleukin
LBD	Large bone defects
luc	Luciferase
µCT	Micro-computed tomography
mL	Milliliter
NF-κB	Nuclear factor “kappa-light-chain-enhancer” of activated B-cells
OC	Osteocalcin
OPG	Osteoprotegerin
Osx	Osterix
PDGF	Platelet-derived growth factor
pg	Picogram
ROI	Region of interest
Sr	Strontium
TNF-α	Tumor necrosis factor-alpha
VEGF	Vascular endothelial growth factor
VEGFR	Vascular endothelial growth factor receptor
